# Supplementary material for: Melittin-lipid nanoparticles target to lymph nodes and elicit a systemic anti-tumor immune response
Source: Nat Commun. 2020 Feb 28;11:1110. doi: 10.1038/s41467-020-14906-9 (PMC7048802; doi:10.1038/s41467-020-14906-9)
Supplement: Supplementary file 1 — Supplementary Information [file 41467_2020_14906_MOESM1_ESM.pdf]

Supplementary Materials for

Melittin-lipid nanoparticles target to lymph node and elicit a systemic anti-tumor immune response

Yu. X. et al

This PDF file includes:

- Supplementary Figure 1: Fluorescence images of the biodistribution of the α-melittin-NPs.
- Supplementary Figure 2: Peripheral blood smears indicate schistocytes in mice treated with melittin.
- Supplementary Figure 3: ALN localization of α-melittin-NPs with macrophages and DCs.
- Supplementary Figure 4: α-melittin-NPs induce the activation of APCs in ALN.
- Supplementary Figure 5: Evaluation of cell viability by MTS assay.
- Supplementary Figure 6: Zeta potential measurement of B16F10 cells, BMDCs and BMDMs.
- Supplementary Figure 7: Evaluation of cellular-binding ability of α-melittin-NPs.
- Supplementary Figure 8: Estimation of cholesterol content.
- Supplementary Figure 9: α-melittin-NPs induce the necrosis/apoptosis of tumor cells and the release of endogenous tumor antigens in vivo.
- Supplementary Figure 10: The photo images of mice after α-melittin-NP therapy.
- Supplementary Figure 11: α-melittin-NPs are mainly restricted to the injected tumor and tumor-draining LNs.
- Supplementary Figure 12: α-melittin-NPs provide durable protection from tumor rechallenge.
- Supplementary Figure 13: Antigen-specific T cells and antibody responses at day 14.
- Supplementary Figure 14: Gating strategy used in flow cytometry analysis.

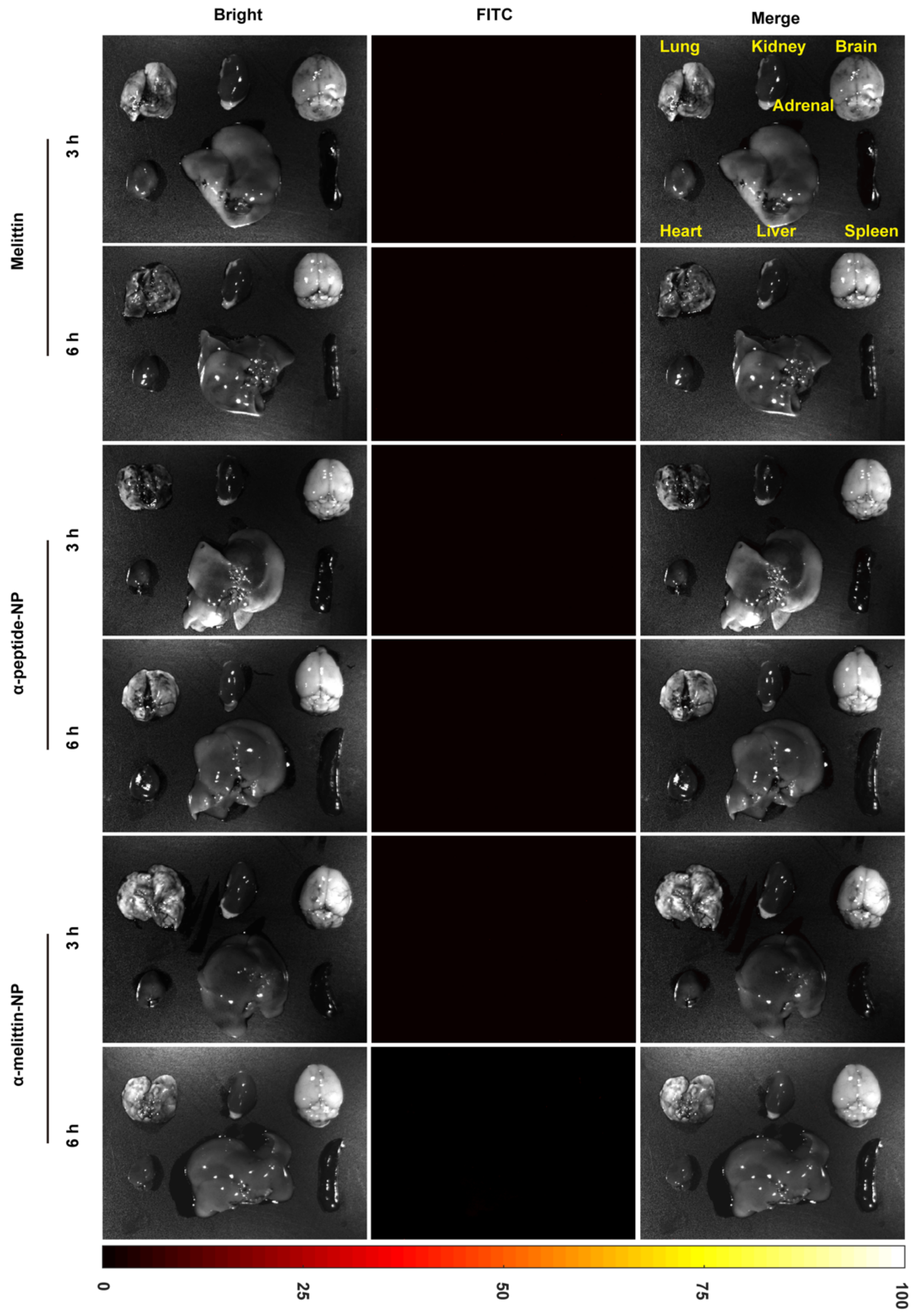

Supplementary Figure 1: Fluorescence images of the biodistribution of α-melittin-NPs. Related to Fig. 1, mice (n = 4 per group) were dissected and organs were collected at 3 h and 6 h after s.c. injection with 20 nmol of FITC-melittin, FITC-α-peptide-NPs, and FITC-α-melittin-NPs (quantification was based on the FITC content). Fluorescence imaging of FITC was acquired with a filter set (excitation: 469/35 nm; emission: 559/34 nm) and representative images from three independent experiments are shown above.

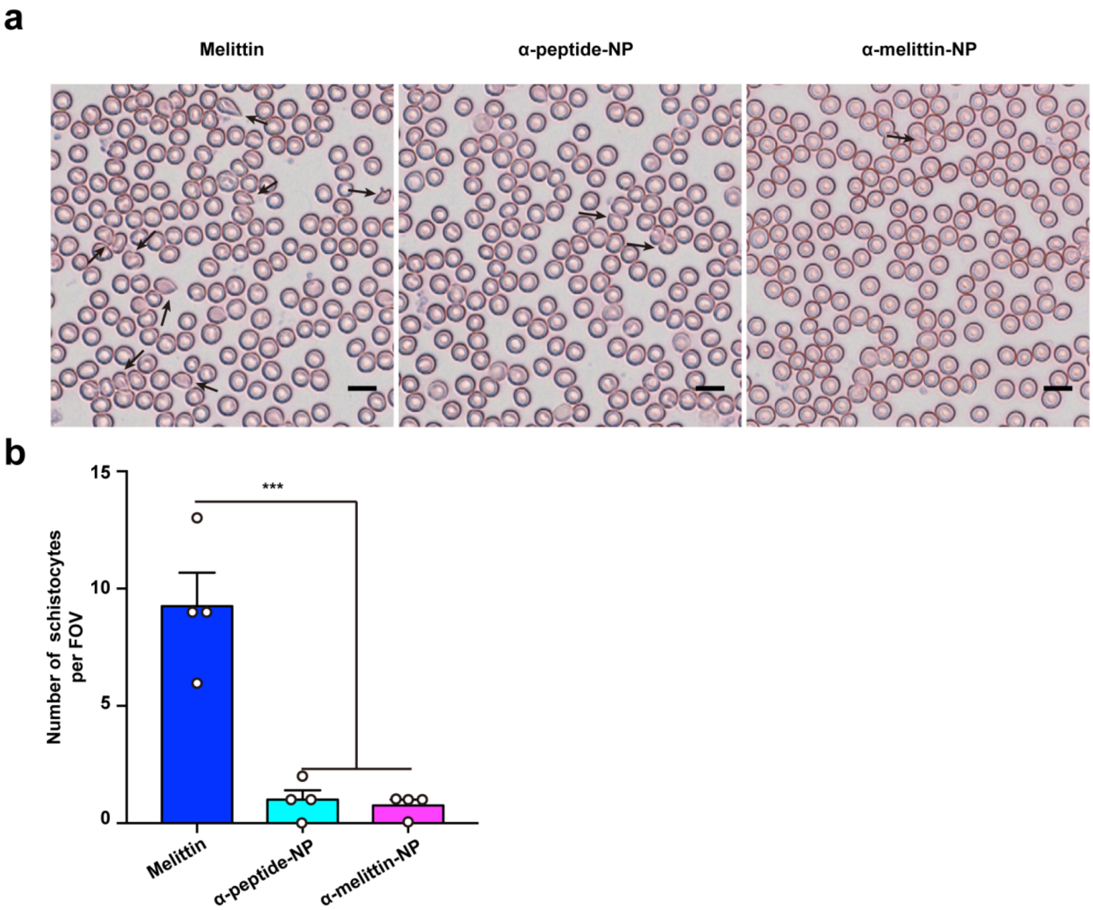

Supplementary Figure 2: Peripheral blood smears contain schistocytes in mice treated with melittin. (**a**, **b**) Mice (n = 4 per group) were injected subcutaneously with 20 nmol of FITC-melittin, FITC-α-peptide-NPs, and FITC-α-melittin-NPs (quantification was based on the FITC content). After 3 h, the blood was collected to prepare peripheral blood smears. Representative images from two independent experiments are shown (**a**). The black arrows indicate the appearance of schistocytes. Scale bar, 10 μm. The number of schistocytes was counted manually from 4 fields of view (FOVs) and are shown (**b**). The size of the FOV is 161.1 μm × 161.1 μm. Data are shown as the mean ± SEM. n.s. not significant, $^{***}P < 0.001$, as analyzed by one-way ANOVA with Bonferroni's post hoc test (**b**). Source data are provided as a Source Data file.

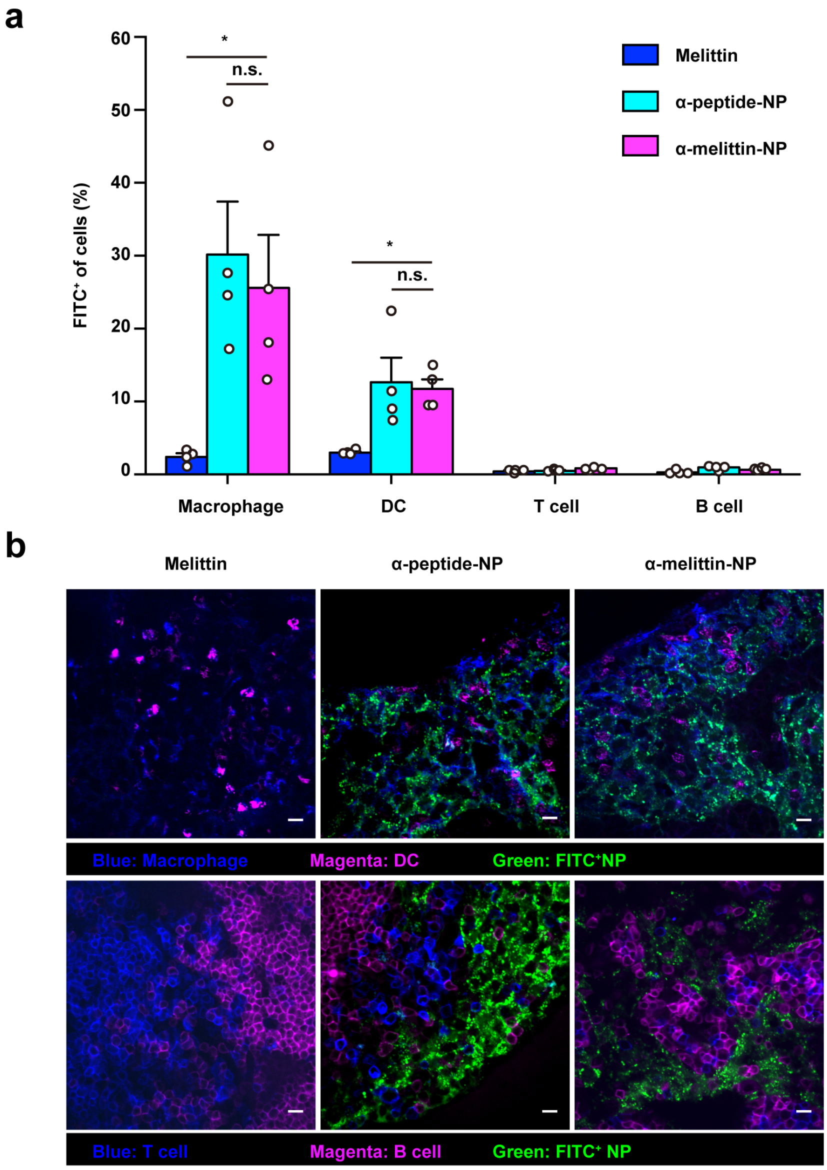

Supplementary Figure 3: ALN localization of α-melittin-NPs with macrophages and DCs. (**a**, **b**) Mice (n = 4 per group) were injected subcutaneously with 20 nmol of FITC-melittin, FITC-α-peptide-NPs, and FITC-α-melittin-NPs (quantification was based on the FITC content). After 3 h, ALNs were sectioned or digested to analyze the localization of melittin, α-peptide-NPs, and α-melittin-NPs in macrophages and DCs. Percentages (**a**) of $FITC^+$ cells in the ALNs and representative immunofluorescence images (**b**) from three independent experiments are shown. Blue and magenta represent $F4/80^+$ macrophages and $CD11c^+$ DCs, respectively, in upper panel and represent $CD3^+$ T cells and $B220^+$ B cells, respectively, in lower panel. Green represents FITC-labelled melittin, α-peptide-NPs, and α-melittin-NPs. Scale bar, 10 μm. Data are shown as the mean ± SEM. n.s. not significant, $^*P < 0.05$, as analyzed by one-way ANOVA with Bonferroni's post hoc test (**a**). Source data are provided as a Source Data file.

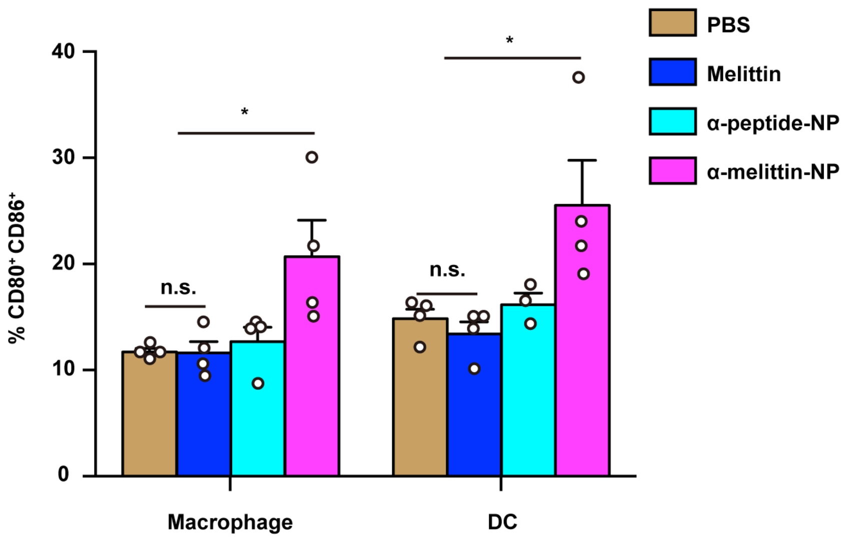

Supplementary Figure 4: α-melittin-NPs induce the activation of APCs in ALNs. Related to Fig. 1, ALNs (n = 4 per group) were collected and digested 24 h after s.c. injection with 35 nmol of melittin, α-peptide-NPs, and α-melittin-NPs (quantification was based on the peptide content). CD80 and CD86 expression levels on macrophages and DCs in ALNs were analyzed using flow cytometry. Data are shown as the mean ± SEM. n.s. not significant, *$P < 0.05$, as analyzed by one-way ANOVA with Bonferroni's post hoc test. Source data are provided as a Source Data file.

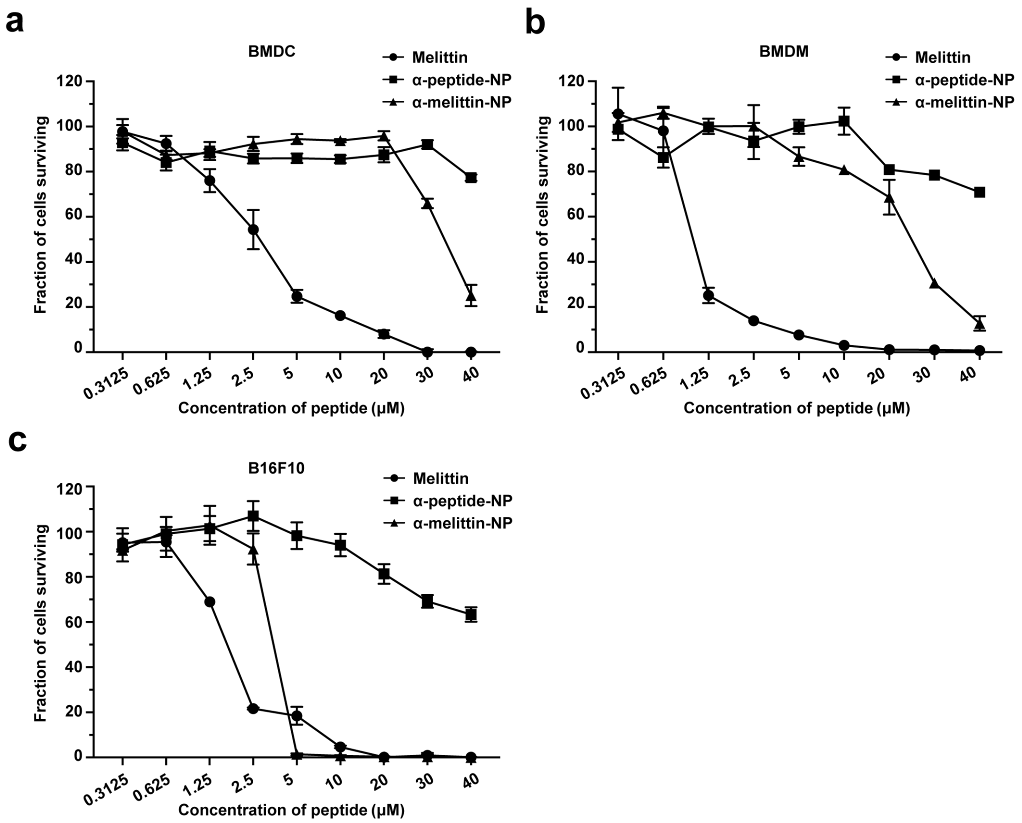

Supplementary Figure 5: Evaluation of cell viability by MTS assay. BMDCs (**a**), BMDMs (**b**) and B16F10 cells (**c**), incubation time: 24 h. Data are shown as the mean ± SEM (n = 3). Source data are provided as a Source Data file.

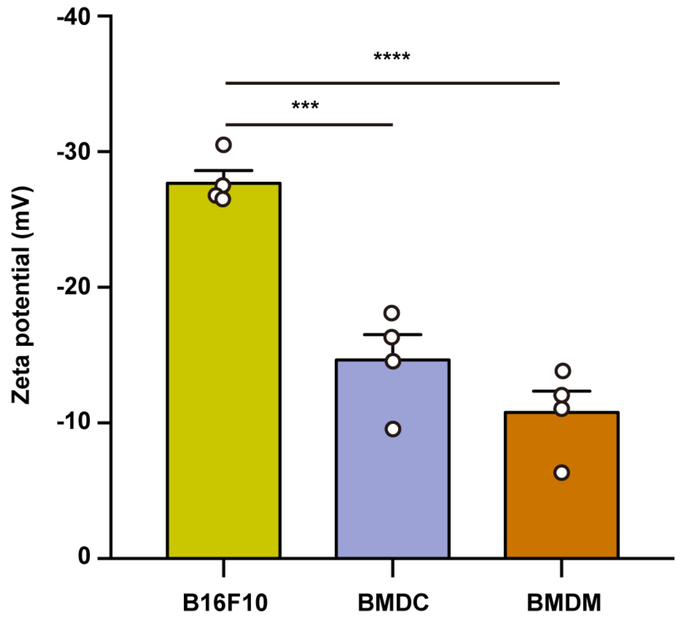

Supplementary Figure 6: Zeta potential measurement of B16F10 cells, BMDCs and BMDMs. Cell membrane potentials of B16F10 cells, BMDCs and BMDMs (n = 4 per group) were measured using a Malvern Zetasizer Nano ZS90 instrument. Data are shown as the mean ± SEM. ***$P < 0.001$ and ****$P < 0.0001$, as analyzed by one-way ANOVA with Bonferroni's post hoc test. Source data are provided as a Source Data file.

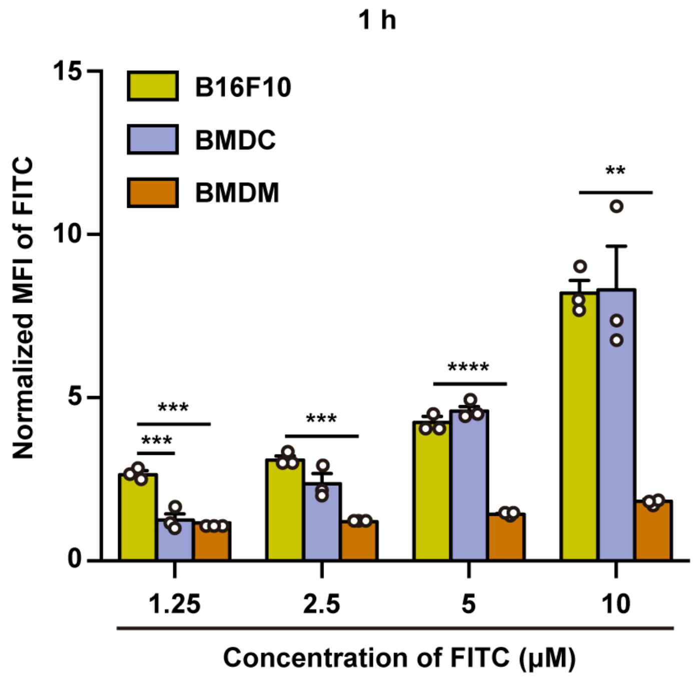

Supplementary Figure 7: Evaluation of cellular-binding ability of α-melittin-NPs. Quantitative data of the mean fluorescent intensity (MFI) of FITC-α-melittin-NPs in B16F10 cells, BMDCs and BMDMs (n = 3 per group). The MFI values were normalized according to minimum (0 μM) in each type of cell. Incubation time: 1 h. Data are shown as the mean ± SEM. **$P < 0.01$, ***$P < 0.001$ and ****$P < 0.0001$, as analyzed by one-way ANOVA with Bonferroni's post hoc test. Source data are provided as a Source Data file.

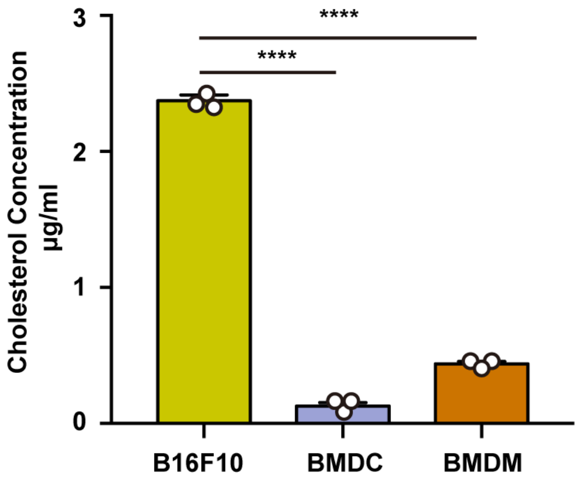

Supplementary Figure 8: Estimation of cholesterol content. B16F10 cells, BMDMs and BMDCs (n = 3 per group) were lysed in PBS containing 2% Triton X-100 for 10 min. After centrifugation (12000 rpm, 15 min), the resulting supernatant was used for detecting cholesterol content with an Amplex® Red reagent–based assay. Data are shown as the mean ± SEM. ****$P < 0.0001$, as analyzed by one-way ANOVA with Bonferroni's post hoc test. Source data are provided as a Source Data file.

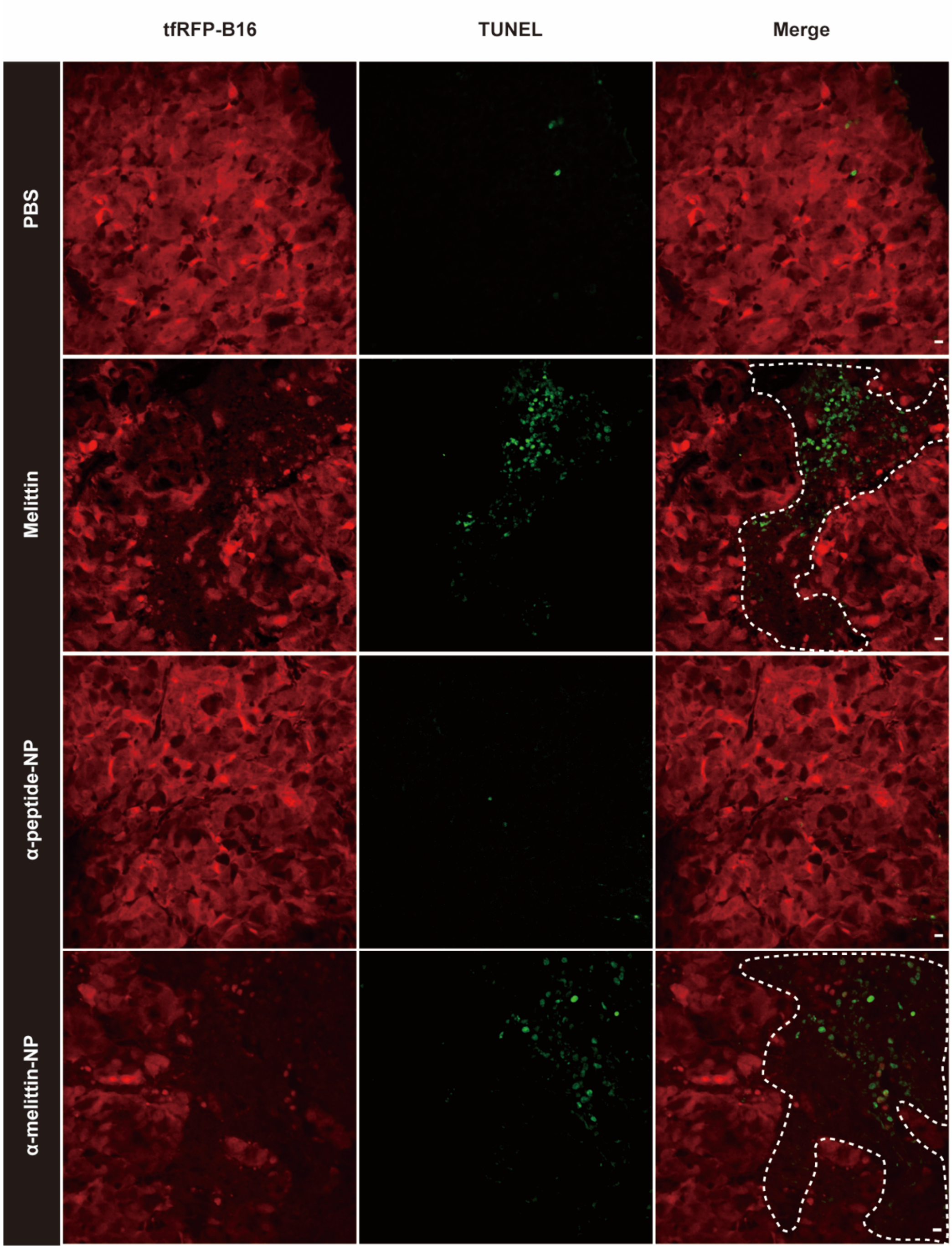

Supplementary Figure 9: α-melittin-NPs induce the necrosis/apoptosis of tumor cells and the release of endogenous tumor antigens in vivo. Mice (n = 3 per group) were inoculated in the left flank with tfRFP-B16F10 cells and treated with intratumoral injections of 35 nmol α-melittin-NPs (quantification was based on the peptide content). Representative immunofluorescence images from three independent experiments are shown above. The white dotted outline indicates the area of necrosis/apoptosis. Scale bar, 10 μm.

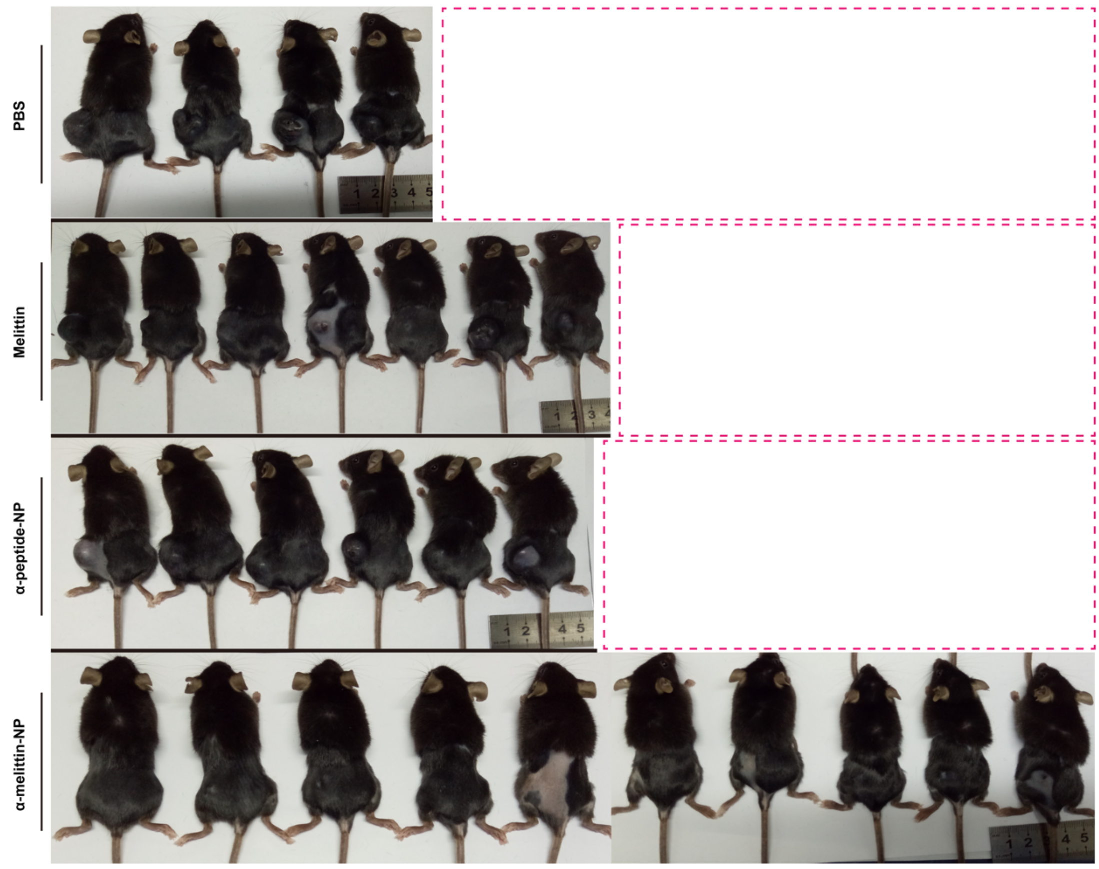

Supplementary Figure 10: The photo images of mice after α-melittin-NP therapy. Related to Fig. 3, B16F10 cells were implanted into the left and right flanks of mice (n = 10 per group) on days 0 and 4, respectively. The mice were treated with intratumoral injections of 35 nmol of melittin, α-peptide-NPs, and α-melittin-NPs (quantification was based on the peptide content) on days 7 and 9. At 20 days post left tumor inoculation, images of mice were taken and shown above. The dotted box indicates the dead mice in the PBS, melittin, and α-peptide-NP groups.

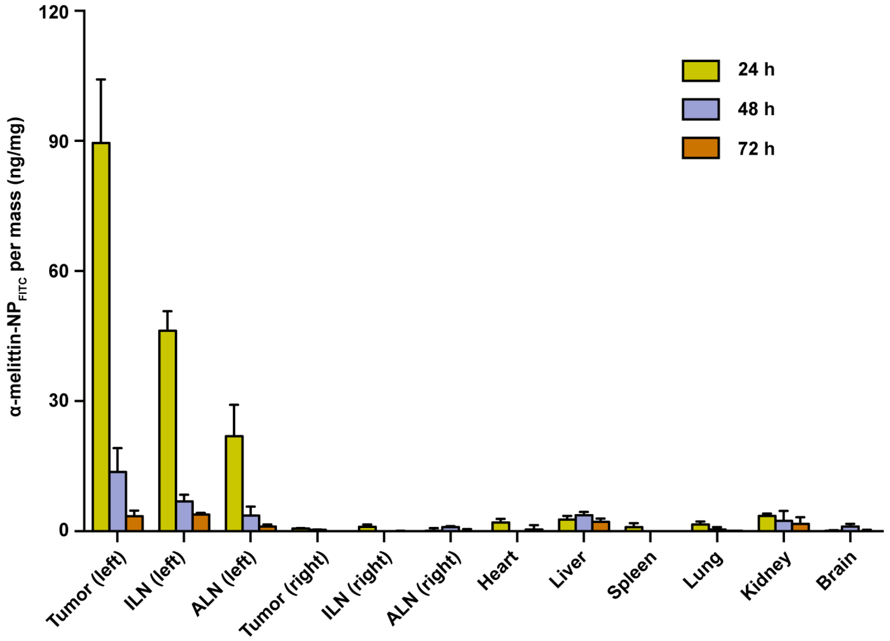

Supplementary Figure 11: α-melittin-NPs are mainly restricted to the injected tumor and tumor-draining LNs. Mice (n = 3 per group) were inoculated with B16F10 cells in the left and right flanks on days 0 and 4, respectively. 20 nmol of FITC-α-melittin-NPs (quantification was based on the FITC content) were intratumoral administered when the tumor size reached 100 mm^3. Tumors and other organs were collected, weighed, and mechanically digested in PBS for 5 mins. Then, the tissues were sonicated using a sonicator for 30 seconds at 3 watts of output power. Following the addition of 10% trichloro-acetic acid in methanol, samples were centrifuged at 12,000 g for 15 min. The detection of FITC in supernatants was performed via fluorescence (excitation: 495 nm; emission: 525 nm) using a microplate reader. Data are shown as the mean ± SEM. Source data are provided as a Source Data file.

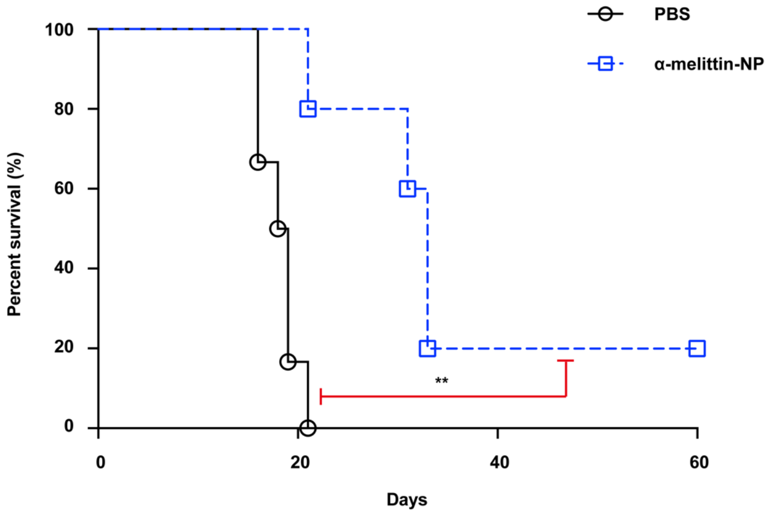

Supplementary Figure 12: α-melittin-NPs provide durable protection from tumor rechallenge. Mice cured of B16F10 melanoma by α-melittin-NPs were injected on day 65 with 1×10^5 B16F10 cells, monitored for tumor growth, and euthanized when the tumors reached 1000 mm^3. Overall animal survival of the PBS group (n = 6) and the α-melittin-NP treatment group (n = 5) are shown. $^{**}P < 0.01$ analyzed by log-rank Mantel–Cox test. Source data are provided as a Source Data file.

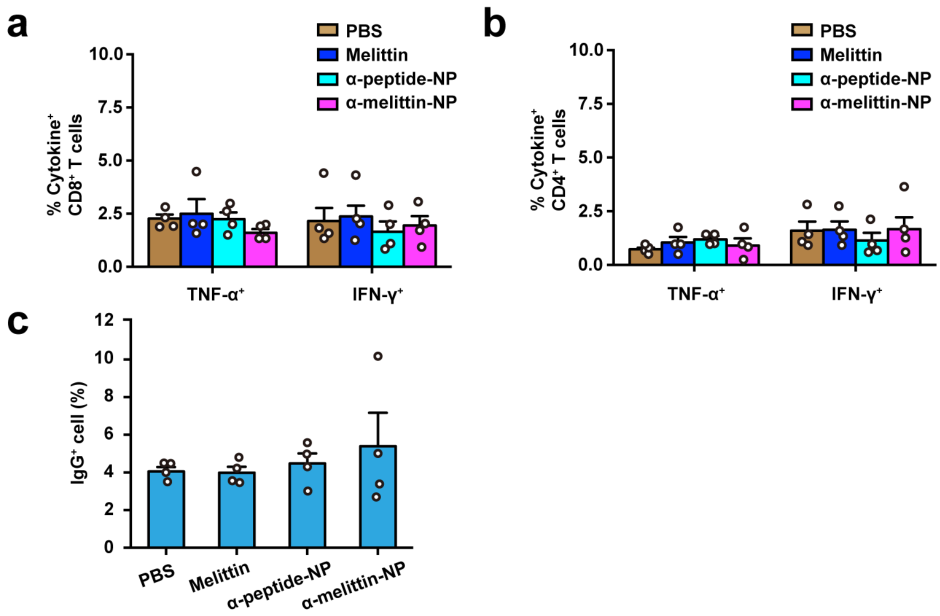

Supplementary Figure 13: Antigen-specific T cells and antibody responses at day 14. (**a**, **b**) C57BL/6 mice (n = 4 per group) were treated as described above (Fig. 3a). On day 14, the lymphocytes isolated from the tumor-draining LNs were restimulated with DCs pulsed with B16F10 tumor lysates and were analyzed by flow cytometry with intracellular cytokine staining. Cytokine^{+} cell frequencies from each group are shown. (**c**) B16F10 tumor cells were incubated with 5% serum that was collected from treated mice and age-matched naïve mice (n = 4 per group). Subsequently, these cells were stained with a DyLight649-conjugated mouse IgG-specific secondary antibody and analyzed by flow cytometry. IgG^{+} cell frequencies from each group are shown. Data are shown as the mean ± SEM. Statistical analysis was performed with one-way ANOVA by Bonferroni's post hoc test (**a-c**). Source data are provided as a Source Data file.

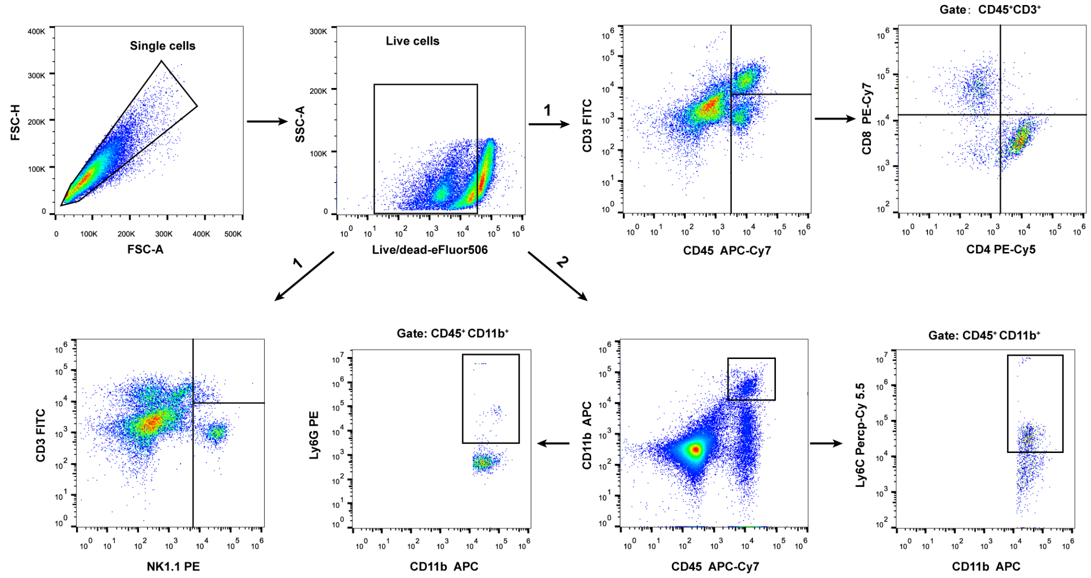

Supplementary Figure 14: Gating strategy used in flow cytometry analysis. Cell clumps are eliminated by FSC-H vs FSC-A gating and live single cells are identified based on the exclusion of a Live/Dead dye (eFluor506). Then samples are divided into two parts, one for detection of the adaptive immune components (Fig. 6b), and the other is used to analyze the innate components (Fig. 6c).
