# Supplementary material for: Melittin-lipid nanoparticles target to lymph nodes and elicit a systemic anti-tumor immune response
Source: Nat Commun. 2020 Feb 28;11:1110. doi: 10.1038/s41467-020-14906-9 (PMC7048802; doi:10.1038/s41467-020-14906-9)
Supplement: Supplementary file 3 — Reporting Summary [file 41467_2020_14906_MOESM3_ESM.pdf]

nature research

Corresponding author(s): Zhihong Zhang

Last updated by author(s): Jan 6, 2020

Reporting Summary

Nature Research wishes to improve the reproducibility of the work that we publish. This form provides structure for consistency and transparency in reporting. For further information on Nature Research policies, see Authors & Referees and the Editorial Policy Checklist .

Statistics

For all statistical analyses, confirm that the following items are present in the figure legend, table legend, main text, or Methods section.

n/a	Confirmed	
☐	☒	The exact sample size (n) for each experimental group/condition, given as a discrete number and unit of measurement
☐	☒	A statement on whether measurements were taken from distinct samples or whether the same sample was measured repeatedly
☐	☒	The statistical test(s) used AND whether they are one- or two-sided<br>*Only common tests should be described solely by name; describe more complex techniques in the Methods section.*
☒	☐	A description of all covariates tested
☐	☒	A description of any assumptions or corrections, such as tests of normality and adjustment for multiple comparisons
☐	☒	A full description of the statistical parameters including central tendency (e.g. means) or other basic estimates (e.g. regression coefficient) AND variation (e.g. standard deviation) or associated estimates of uncertainty (e.g. confidence intervals)
☐	☒	For null hypothesis testing, the test statistic (e.g. F, t, r) with confidence intervals, effect sizes, degrees of freedom and P value noted<br>*Give P values as exact values whenever suitable.*
☒	☐	For Bayesian analysis, information on the choice of priors and Markov chain Monte Carlo settings
☒	☐	For hierarchical and complex designs, identification of the appropriate level for tests and full reporting of outcomes
☒	☐	Estimates of effect sizes (e.g. Cohen's d, Pearson's r), indicating how they were calculated

Our web collection on statistics for biologists contains articles on many of the points above.

Software and code

Policy information about availability of computer code

Data collection	The Volocity version 6.3 was used to collect the intravital imaging data; the CytExpert software version 2.3 and micro-capillary flow cytometer was used to collect the flow cytometry data; the NIS-Elements software version 3.20 was used to collect the HE image data and morphology data of RBCs; the Zeiss LSM 710 confocal imaging system was used to collect the tissue sections and fixed cells imaging data; the UNICORN version 5.11 was used to collect the data about synthesis of nanoparticle; the Zetasizer software version 7.13 was used to collect the zeta potentials data.
Data analysis	All fluorescence images were analyzed with Image J software version 1.52; The flow cytometry data were analyzed with FlowJo software version 10.7; Cytokine/chemokine quantitation was analyzed with LEGENDplex software version 8.0. All statistical analyses were performed with GraphPad Prism software version 7.2.

For manuscripts utilizing custom algorithms or software that are central to the research but not yet described in published literature, software must be made available to editors/reviewers. We strongly encourage code deposition in a community repository (e.g. GitHub). See the Nature Research guidelines for submitting code & software for further information.

Data

Policy information about availability of data

All manuscripts must include a data availability statement. This statement should provide the following information, where applicable:
- Accession codes, unique identifiers, or web links for publicly available datasets
- A list of figures that have associated raw data
- A description of any restrictions on data availability

The authors declare that the main data supporting the findings of this study are available within the article and its Supplementary Materials. Extra data are available from the corresponding author upon reasonable request. The source data underlying Figs. 1c, d, g, 2d, 3c-f, 4b, c, 5b-e and 6b, c, e and Supplementary Figs. 2b, 3a, 4, 5a-c, 6-8, 11-12, and 13a-c are provided with the paper as a Source Data file.

Field-specific reporting

Please select the one below that is the best fit for your research. If you are not sure, read the appropriate sections before making your selection.

- [x] Life sciences
- [ ] Behavioural & social sciences
- [ ] Ecological, evolutionary & environmental sciences

For a reference copy of the document with all sections, see nature.com/documents/nr-reporting-summary-flat.pdf

Life sciences study design

All studies must disclose on these points even when the disclosure is negative.

Sample size	No sample size calculation was performed. Sample size was determined to be adequate based on the magnitude and consistency of measurable differences between groups. In addition,the sample sizes of this study were determined on the basis of similar published studies. In anti-tumor experiments, 10 mice each group were used for long time monitoring of tumor volume, 10 mice each group were used to verify the specific systemic response, 6 mice in a-meliitin-NP group were used to determine the durable antitumor response. In vitro experiments, the sample size for each group was 3-4.
Data exclusions	No data were excluded from the analyses in the study.
Replication	Every experiment was repeated once or more to ensure every major finding is highly repeatable.
Randomization	To prevent selection bias, mice (female, 6-8 weeks, n=10 per group) were randomly allocated to different groups before treatment.
Blinding	The investigators were not blinded to group allocation during data collection except some data (HE, Cholesterol estimation, immunofluorescence staining). Before the experiment were performed, we have to check the study procedure and analysis methods were correctly done. However, the treatment efficacy was apparent in both quantification and representative images of outcomes.

Reporting for specific materials, systems and methods

We require information from authors about some types of materials, experimental systems and methods used in many studies. Here, indicate whether each material, system or method listed is relevant to your study. If you are not sure if a list item applies to your research, read the appropriate section before selecting a response.

Materials & experimental systems

n/a	Involved in the study	
[ ]	[x]	Antibodies
[ ]	[x]	Eukaryotic cell lines
[x]	[ ]	Palaeontology
[ ]	[x]	Animals and other organisms
[x]	[ ]	Human research participants
[x]	[ ]	Clinical data

Methods

n/a	Involved in the study	
[x]	[ ]	ChIP-seq
[ ]	[x]	Flow cytometry
[x]	[ ]	MRI-based neuroimaging

Antibodies

Antibodies used	Biolegend: CD45 (Clone: 30-F11, Catalog: 103116/103112/103108), CD3 (Clone: 145-2C11/17A2, Catalog: 100308/100204), CD4 (Clone: RM4-5, Catalog: 100514), CD8 (Clone: 53-6.7, Catalog: 100722), B220 (Clone: RA3-6B2, Catalog: 103223), CD11c (Clone: N418, Catalog: 117316/117324), F4/80 (Clone: BM8, Catalog: 123132), CD11b (Clone: M1/70, Catalog: 101216/101212), Ly-6G (Clone: 1A8, Catalog: 127608/127624), Ly-6C (Clone: HK1.4, Catalog: 128012), NK1.1 (Clone: PK136, Catalog: 108710), CD80 (Clone: 16-10A1, Catalog: 104722), CD86 (Clone: GL-1, Catalog: 105012), IFN-γ (Clone: XMG1.2, Catalog: 505808), TNF-α (Clone: MP6-XT22, Catalog: 506308) , CD16/32 (Clone: 93, Catalog: 101320),F4/80 (Clone: BM8, Catalog: 123132, 1:200), CD11c (Clone: N418, Catalog: 117314, 1:100), CD3 (Clone: 17A2, Catalog: 100240, 1:200), B220 (Clone: RA3-6B2, Catalog: 103226, 1:200), and CD8 (Clone: 53-6.7, Catalog: 100724, 1:200).<br>BD Biosciences:CD4 (Catalog: 562891, 1:200).<br>eBioscience: Fixable viability dye eFluor506 (Catalog: 65-0866-18).
Validation	CD45, Catalog: 103116, Isotype: Rat IgG2b, κ, Application: FC (Flow Cytometry).The validation has been performed by the manufacturer:https://www.biolegend.com/en-us/products/apc-cyanine7-anti-mouse-cd45-antibody-2530.<br>CD45, Catalog: 103112,Isotype: Rat IgG2b, κ,Application: FC (Flow Cytometry).The validation has been performed by the manufacturer: https://www.biolegend.com/en-us/products/apc-anti-mouse-cd45-antibody-97.<br>CD45, Catalog: 103108,Isotype: Rat IgG2b, κ, Application: FC (Flow Cytometry).The validation has been performed by the manufacturer:https://www.biolegend.com/en-us/products/fitc-anti-mouse-cd45-antibody-99.<br>CD3, Catalog: 100308,Isotype: Armenian Hamster IgG,Application: FC (Flow Cytometry).The validation has been performed by the manufacturer:https://www.biolegend.com/en-us/products/pe-anti-mouse-cd3epsilon-antibody-25.

CD3, Catalog: 100204,Isotype:Rat IgG2b, κ, Application: FC (Flow Cytometry).The validation has been performed by the manufacturer:https://www.biolegend.com/en-us/products/fitc-anti-mouse-cd3-antibody-45
CD4,Catalog: 100514,Isotype:Rat IgG2a, κ,Application: FC (Flow Cytometry).The validation has been performed by the manufacturer:https://www.biolegend.com/en-us/products/pe-cy5-anti-mouse-cd4-antibody-483
CD8,Catalog: 100722,Isotype:Rat IgG2a, κ,Application: FC (Flow Cytometry). The validation has been performed by the manufacturer:https://www.biolegend.com/en-us/products/pe-cy7-anti-mouse-cd8a-antibody-1906
B220,Catalog: 103223,Isotype:Rat IgG2a, κ, Application: FC (Flow Cytometry).The validation has been performed by the manufacturer:https://www.biolegend.com/en-us/products/apc-cy7-anti-mouse-human-cd45r-b220-antibody-1938
CD11c,Catalog: 117316,Isotype: Armenian Hamster IgG, Application: FC (Flow Cytometry).The validation has been performed by the manufacturer:https://www.biolegend.com/en-us/products/pe-cy5-anti-mouse-cd11c-antibody-3085
CD11c,Catalog: 117324,Isotype: Armenian Hamster IgG, Application: FC (Flow Cytometry).The validation has been performed by the manufacturer:https://www.biolegend.com/en-us/products/apccyanine7-anti-mouse-cd11c-antibody-3931
F4/80, Catalog: 123132,Isotype: Rat IgG2a, κ, Application: FC (Flow Cytometry),IHC-F (Immunodetection applied to frozen tissues).The validation has been performed by the manufacturer:https://www.biolegend.com/en-us/products/brilliant-violet-421-anti-mouse-f4-80-antibody-7199
CD11b, Catalog: 101216,Isotype: Rat IgG2b, κ, Application: FC (Flow Cytometry).The validation has been performed by the manufacturer:https://www.biolegend.com/en-us/products/pe-cy7-anti-mouse-human-cd11b-antibody-1921
CD11b, Catalog: 101212,Isotype: Rat IgG2b, κ, Application: FC (Flow Cytometry).The validation has been performed by the manufacturer:https://www.biolegend.com/en-us/products/apc-anti-mouse-human-cd11b-antibody-345
Ly-6G, Catalog: 127608,Isotype: Rat IgG2a, κ, Application: FC (Flow Cytometry).The validation has been performed by the manufacturer:https://www.biolegend.com/en-us/products/pe-anti-mouse-ly-6g-antibody-4777
Ly-6G, Catalog: 127624,Isotype: Rat IgG2a, κ, Application: FC (Flow Cytometry).The validation has been performed by the manufacturer:https://www.biolegend.com/en-us/products/apc-cy7-anti-mouse-ly-6g-antibody-6755
CD86, Catalog: 105012,Isotype: Rat IgG2a, κ, Application: FC (Flow Cytometry).The validation has been performed by the manufacturer:https://www.biolegend.com/en-us/products/apc-anti-mouse-cd86-antibody-2896
Ly-6C, Catalog: 128012,Isotype: Rat IgG2c, κ, Application: FC (Flow Cytometry).The validation has been performed by the manufacturer:https://www.biolegend.com/en-us/products/percp-cy5-5-anti-mouse-ly-6c-antibody-5967
NK1.1, Catalog: 108710,Isotype: Mouse IgG2a, κ, Application: FC (Flow Cytometry).The validation has been performed by the manufacturer:https://www.biolegend.com/en-us/products/apc-anti-mouse-nk-1-1-antibody-427
CD80, Catalog: 104722,Isotype: Armenian Hamster IgG, Application: FC (Flow Cytometry).The validation has been performed by the manufacturer:https://www.biolegend.com/en-us/products/percpcyanine55-anti-mouse-cd80-antibody-4275
IFN-γ, Catalog: 505808,Isotype: Rat IgG1, κ, Application: ICFC (Flow cytometric analysis of intracellularly-stained cells).The validation has been performed by the manufacturer:https://www.biolegend.com/en-us/products/pe-anti-mouse-ifn-gamma-antibody-997
TNF-α , Catalog: 506308,Isotype: Rat IgG1, κ, Application: ICFC (Flow cytometric analysis of intracellularly-stained cells).The validation has been performed by the manufacturer:https://www.biolegend.com/en-us/products/apc-anti-mouse-tnf-alpha-antibody-975
CD16/32, Catalog: 101320,Isotype: Rat IgG2a, λ, Application: FC (Flow Cytometry).The validation has been performed by the manufacturer:https://www.biolegend.com/en-us/products/trustain-fcx-anti-mouse-cd16-32-antibody-5683
F4/80, Catalog: 123132,
CD11c, Catalog: 117314,Isotype: Armenian Hamster IgG, Application: FC (Flow Cytometry) ,IHC(Analysis of antibody-labeled tissue preparations), IF(Microscopic examination of fluorescently-labeled cells).The validation has been performed by the manufacturer:https://www.biolegend.com/en-us/products/alexa-fluor-647-anti-mouse-cd11c-antibody-2703
CD3, Catalog: 100240,Isotype: Rat IgG2b, κ, Application: FC (Flow Cytometry) ,IHC(Analysis of antibody-labeled tissue preparations), IHC-F(Immunodetection applied to frozen tissues).The validation has been performed by the manufacturer:https://www.biolegend.com/en-us/products/alexa-fluor-594-anti-mouse-cd3-antibody-9735
B220, Catalog: 103226, Isotype: Rat IgG2a, Application: FC (Flow Cytometry) ,IHC(Analysis of antibody-labeled tissue preparations), IHC-F(Immunodetection applied to frozen tissues).The validation has been performed by the manufacturer:https://www.biolegend.com/en-us/products/alexa-fluor-647-anti-mouse-human-cd45r-b220-antibody-2708
CD4, Catalog: 562891,Isotype: Rat LEW, Application: Flow cytometry (Routinely Tested) ,Immunofluorescence (Tested During Development).The validation has been performed by the manufacturer:https://www.bdbiosciences.com/cn/applications/research/t-cell-immunology/th-1-cells/surface-markers/mouse/bv421-rat-anti-mouse-cd4-gk15/p/562891.
CD8, Catalog: 100724,Isotype: Rat IgG2a, Application: FC (Flow Cytometry) ,IHC(Analysis of antibody-labeled tissue preparations), IHC-F(Immunodetection applied to frozen tissues).The validation has been performed by the manufacturer:https://www.biolegend.com/en-us/products/alexa-fluor-647-anti-mouse-cd8a-antibody-2699
Fixable viability dye eFluor506, Catalog: 65-0866-18, Application: FC (Flow Cytometry).The validation has been performed by the manufacturer:https://www.thermofisher.com/order/catalog/product/65-0866-18?SID=srch-hj-65-0866-18

Eukaryotic cell lines

Policy information about cell lines

Cell line source(s)

The B16F10 cell line was purchased from the BOSTER Company (Wuhan, China). The E0771 cell line was kindly provided by Rong Xiang (Medical School of Nankai University, Tianjin, China) and were authenticated using short tandem repeat (STR) profiling.

Authentication

These cell lines were authenticated using short tandem repeat (STR) profiling.

Mycoplasma contamination

These cell lines were mycoplasma-negative as determined by screening using the MycoProbe Mycoplasma Detection Kit (R and D Systems, Minneapolis, MN).

Commonly misidentified lines (See ICLAC register)	There are no commonly misidentified cell lines in the study.

Animals and other organisms

Policy information about studies involving animals; ARRIVE guidelines recommended for reporting animal research

Laboratory animals	Wild-type C57BL/6 mice (Female, 6-8 weeks old) .<br>Actb-EGFP C57BL/6 mice (Female, 6-8 weeks old).<br>mT/mG transgenic mice (Female, 6-8 weeks old).<br>All of the mice were bred and maintained in a specific pathogen-free (SPF) barrier facility at Animal Center of Wuhan National Laboratory for Optoelectronics.
Wild animals	The study did not involve wild animals.
Field-collected samples	The study did not involve samples collected from the field.
Ethics oversight	All animal studies were conducted in compliance with protocols that had been approved by the Hubei Provincial Animal Care and Use Committee and in compliance with the experimental guidelines of the Animal Experimentation Ethics Committee of Huazhong University of Science and Technology.

Note that full information on the approval of the study protocol must also be provided in the manuscript.

Flow Cytometry

Plots

Confirm that:

- [x] The axis labels state the marker and fluorochrome used (e.g. CD4-FITC).
- [x] The axis scales are clearly visible. Include numbers along axes only for bottom left plot of group (a 'group' is an analysis of identical markers).
- [x] All plots are contour plots with outliers or pseudocolor plots.
- [x] A numerical value for number of cells or percentage (with statistics) is provided.

Methodology

Sample preparation	Cell were isolated from lymph node and tumor as follow: lymph node and tumors were removed using forceps and surgical scissors and weighed. Then they were minced with scissors prior to incubation with 2 mg/mL collagenase IV and 0.2 mg/mL DNase I (Sigma-Aldrich) for 30 min at 37℃. These tissues were homogenized by repeated pipetting and filtered through a 70 µm cell strainer, and then washed once with complete RPMI to prepare a single cell suspension.
Instrument	CytoFLEX flow cytometer (Beckman Coulter, USA).
Software	The experiment data were collected by CytExpert software version 2.3 (Beckman Coulter, USA) and analyzed by FlowJo software (FlowJo, Ashland, OR, USA).
Cell population abundance	No sorting was involved.
Gating strategy	Cell clumps are eliminated by FSC-H vs FSC-A gating and live single cells are identified based on the exclusion of a Live/Dead dye (eFluor506). Then samples are divided into two parts, one for detection of the adaptive immune components, and the other is used to analyze the innate components.Conventional innate and adaptive immune components were gated by corresponding marks.

- [x] Tick this box to confirm that a figure exemplifying the gating strategy is provided in the Supplementary Information.
